# Editorial: Analyzing and computing humans - the role of language, culture, brain and health

**DOI:** 10.3389/fnhum.2024.1439729

**Published:** 2024-07-02

**Authors:** Cornelia Herbert, Georg Northoff

**Affiliations:** ^1^Applied Emotion and Motivation Psychology, Institute of Psychology and Education, Faculty of Engineering, Computer Science and Psychology, Ulm University, Ulm, Germany; ^2^Institute of Mental Health Research, University of Ottawa, Ottawa, ON, Canada

**Keywords:** brain-computer interfaces (BCI), language, mental health, cultural differences, self-concept, EEG, self-relevance, resting state

## 1 Analyzing and computing humans by technology – monitoring the mind and enabling behavior by brain computer interfaces (BCIs)

Research from various scientific disciplines has investigated the neurobiological and neurophysiological basis of human experience and behavior, cognitive and affective processing, perception, action, and thought, as well as their role in the prediction of mental disorders, health, and wellbeing. Based on this knowledge, new technological developments paved the way for the investigation of human experience and behavior, and their behavioral, neurophysiological, and psychophysiological correlates inside and outside the controlled laboratory setting. The interface of these devices can be equipped with smart sensors and intelligent technology for the automated recording, monitoring, computation, and analysis of biosignals as markers of the user's mental and behavioral traits and states. Biosignal recording can include but is not limited to, the recording of brain activity, cardiac activity, facial expressions, detection of changes in gaze, temperature, or physical activity, to name but a few examples. Whether for self-monitoring of the user's mental activity, mood, or stress level or for the development of computer-aided support of everyday lives of those users who suffer from neurological disorders or for leisure entertainment—the number of possible use cases of automated biosignal recording is constantly increasing and can be as diverse as human life (for an overview see e.g., Aricò et al., 2018; Sajno et al., 2022).

In particular, monitoring changes in brain activity using EEG (electroencephalography) and brain-computer interface technology (BCI) are attracting great interest and benefit from rapid technological progress. Because BCIs link the external and internal world of a user by translating the user's brain activity into adaptive and assistive computer commands, BCIs are an ideal tool and a prominent example of computer-assisted communication and action control. Originally developed as a communication device for severely disabled patient groups (e.g., Daly and Wolpaw, 2008; Chaudhary et al., 2016), BCI research has quickly expanded to other user groups and application domains to support health care in the occupational or educational setting or for rehabilitation purposes or to use BCIs as a thought-translation device for arts, sports or leisure activities of healthy users (for reviews see e.g., Kawala-Sterniuk et al., 2021). According to recent estimates, by 2030, the market for BCIs will increase steadily and globally, meaning that more and more people will reach access to BCIs for ambulatory biosignal recording, BCI-based experience sampling, or other purposes of behavior analysis (https://www.grandviewresearch.com/industry-analysis/brain-computer-interfaces-market).

## 2 Human factors, language and culture in BCI research: challenges for the present and opportunities for the future

The rapid advancements of BCIs is not without challenges and concerns. Research has shown that the successful use of new technologies, including BCIs, heavily depends on the user's mental, cognitive, motivational, and affective traits, states and abilities. Moreover, understanding the users of brain-computer interfaces requires understanding their language, as well as their social, linguistic, and cultural background and embedding. Although there is evidence from neuroscientific research that the human brain and its functional networks are adaptable and influenced by individual differences in personality traits and mental states, language, and culture (e.g., Friederici and Wartenburger, 2010; Kitayama and Uskul, 2011; Kroll et al., 2015), the investigation of the relationship between BCI performance and human factors, including linguistic and cultural aspects, is still in its infancy compared to other BCI research areas and requires further investigation. In particular, defining important human factors among the many factors examined in previous BCI research that significantly influence BCI performance across different BCIs and use cases requires more attention.

Expanding BCI research to answer these questions is important for several reasons. For example, one reason is the increasing number of users worldwide, the second reason is the growing number of use cases, and a third reason is previous research suggesting that a significant number of BCI users are unable to accurately use a BCI, a well-known problem from the literature as BCI illiteracy or BCI inefficiency (e.g., Allison and Neuper, 2010; Edlinger et al., 2015). Therefore, exploring language, cultural differences, and important overarching key human factors from theoretical, ethical, and methodological perspectives should be considered a priority for future BCI research.

## 3 Aim, scope, and structure of the Research Topic

This Research Topic aims to contribute to this endeavor by discussing how technological advances in BCI research might be examined in the context of human factors, language, and cultural differences of their BCI users. The collection of articles in this Research Topic focuses on five main themes, each of which revolves around a specific question.

**Theme 1:** What are the primary human factors examined in previous research and what evidence is there of their influence on BCI performance?

**Theme 2:** What are the key human factors that could improve BCI performance, and how can they be implemented in future BCI designs?

**Theme 3:** Could self-relevance, self-concept, and resting state markers be factors that promote and predict the user's performance in the BCI context?

**Theme 4:** How can brain-computer interface applications accommodate the diverse language competences of their users and adapt to linguistic and cultural differences of their users?

**Theme 5:** What recommendations can be made for the development of brain-computer interfaces that are sensitive to human factors, as well as linguistically and culturally sensitive?

The five main themes, their discussions and the articles supporting them are arranged chronologically in the Research Topic and are briefly summarized and discussed below. For answers to the themes 4 and 5, the interested reader of this Research Topic should also consider the contributions of two joint Research Topics on *Neurocomputational Models of Language Processing*^1^ for a thorough discussion of recent developments in computational language processing and ethical considerations of privacy and ownership including *The ethics of speech ownership in the context of neural control of augmented assistive communication*^2^ that may arise when using self-referential and lingustically and culturally sensitive BCIs.

## 4 Summary of the research contributions of the Research Topic

### 4.1 Themes 1-2: previous human factor research and the search for key human factors

The first section and review article of the Research Topic addresses the first two questions (see theme 1 and theme 2 above) and provides answers to the third question (see theme 3 above). It reviews and discusses what primary human factors have been examined in previous research and how these factors influenced BCI performance. The review places particular focus on BCI studies that examined the relationship between user traits and states and BCI performance. Only those studies are reviewed that used well-controlled experimental environments with similar standardized tasks, tools and questionnaires and that examined healthy or vulnerable BCI user groups in the P300-BCIs or SMR-BCIs that are the most examined types of BCIs so far. By considering the results of these studies together, the review extends excellent and methodologically well-controlled previous reviews that specifically focused on one of the two types of BCIs, P300-BCIs or SMR-BCIs (for an overview see Herbert in this section). The results of the review suggest that several user characteristics can modulate BCI performance in the two types of BCIs. Certain personality traits as well as user states in particular, such as a high current motivation of the user in using a BCI, can have a positive impact on BCI performance among healthy and vulnerable BCI user groups. Conversely, there are traits and states such as empathy, emotional stability, fatigue or high cognitive effort that have been found across studies to negatively influence BCI performance. The large number of user characteristics and states, as well as the cognitive and time effort required to assess them all together using standardized test batteries prior to BCI training, raise the question of the third theme of the Research Topic, whether there are important human factors that influence and predict BCI performance in general.

### 4.2 Theme 3: key human factors

#### 4.2.1 Self-relevance and the user's self-concept as key human factors

Following up on the results of the review, the second part of the review article of this Research Topic aims to identify overarching or superordinate key human factors that could influence BCI performance across different BCI settings, applications and user groups. In summary, two key human factors are proposed that may allow to group the influence of previously examined user characteristics on BCI performance according to two main dimensions or concepts: namely, the self-relevance of tasks and stimuli and the user's self-concept (see Herbert). Previous evidence from the literature is illustrated along with the theoretical conceptualizations of these two constructs. In particular, evidence is provided that shows how information processing, motivation, cognition, affect and attention is influenced by self-relevance and the self-concept of the user, as well as how both, the self-relevance of task and stimuli and the user's self-concept, can influence the user's sense of ownership, agency, and autonomy. Notably, all the aforementioned factors including ownership, agency, and autonomy have proven relevant human factors for the successful use of a BCI in previous studies. Next, recommendations and examples are provided that go beyond the mere demonstration of evidence. In particular, the article concludes with suggestions and illustrations as to how paradigms, user instructions and BCI training could be made self-relevant and tailored to the user's self-concept in future BCI research and applications. The use of self-referential stimuli, tasks and instructions, the use of brief assessment tools for self-concept traits as well as the use of resting state predictors as neurophysiological features or self-directed BCI training (see also in the next sections 4.2.2 and 4.2.3) are just some of the promising examples discussed in the article and in the following contributions of this Research Topic that could increase the self-relevance of a particular BCI application to the user and facilitate the user's motivation, and feeling of ownership and self-control.

#### 4.2.2 Self-paced user training as a key human factor modulating neurofeedback performance

The facilitative effect of self-paced vs. externally controlled training demonstrated in the next study and in previous EEG neurofeedback studies supports the above suggestion of increasing the self-relevance of BCIs. This study (Uslu and Vögele) found in an investigation with a sample of 60 healthy participants that participants who could pace the neurofeedback training on their own and hence, had high self-control over their training showed enhanced cognitive performance and better learning rates compared to the participants who were training in an externally paced manner. In addition, a significant positive relationship was reported between brain activity (upper alpha) during the neurofeedback session and the resting state activity of the participants.

#### 4.2.3 Interindividual differences in resting state activity as neurophysiological trait and state predictors of task-related activity and BCI performance

In particular, and as additionally pointed out in the review article (Herbert), resting state activity has been shown to be a strong predictor of BCI performance (for a discussion e.g., Blankertz et al., 2009). The reasons for this are still being discussed. One of the reasons could be the relationship between resting state activity and sensorimotor activity and specific changes in task- and resting state networks of relevance for BCI performance. In line with this, another reason could be that resting state activity occurs in resting state networks belonging to cortical midline structures (CMS) and the default mode network, whose activity has been shown to be associated with self-referential processing and self-relevance processing (Schneider et al., 2008; Zhang et al., 2023).

In summary, previous studies have provided evidence that brain activity during a resting state is not a state of rest of all and one activity (for overviews and discussions, e.g., Northoff et al., 2010; Deco et al., 2011; Northoff, 2023, 2024). Instead it is characterized by a number of characteristic states of intrinsic brain activity occuring in a number of brain networks. These changes in spontaneous brain activity occur synchronously in certain brain regions and can fluctuate across networks dynamically. The fluctuations among these functional intrinsic connectivity networks can be associated with certain mental states and physiological states (Chen et al., 2020) during rest or they can influence mental processing, cognitive performance or brain activity during task-related activity following the resting state in healthy or vulnerbale individuals (e.g., Northoff et al., 2010; Gupta et al., 2021; Wolff et al., 2022). Several resting state brain networks have been identified that might be hierachically organized and whose intrinsic activity is distinct from or partially overlapping with task-related or task-positive brain networks that change activity when processing or performing a certain task (for an overview e.g., Seitzman et al., 2019).

Therefore, as a neurophysiological marker or neurophysiological user trait, individual differences in resting state activity could explain a significant portion of variance in task-related activity, mental and cognitive performance across BCI training sessions, BCI paradigms and different types of BCIs. The two contributions of the Research Topic compiled chronologically next, follow up on this idea. The two studies demonstrate that social factors such as the user's occupation (Wu et al.) or the use of assistive technologies such as cochlear implants (Koirala et al.) can modulate the brain's resting state activity and predict changes in dedicated brain networks such as the brain's language network. The impact of occupations was examined among seafarers and a control group, and functional magnetic resonance imaging (fMRI) was used by the authors as a method to compare resting state functional connectiviy of the experimental group with the control group. It is noteworthy that this study is consistent with a number of previous studies that demonstrated occupational neuroplasticity (for a review see Wu et al., 2020). The scope of BCI applications as support tool continues to expand, and includes already many use cases of cognitive control in occupational or educational contexts (for reviews e.g., Belo et al., 2021; Xia et al., 2023). Therefore, findings such as those on occupational or possibly educational and developmental neuroplasticity need to be considered in future BCI research to avoid BCI inefficiency in the long term. In support of this suggestion, the second resting state study of this Research Topic that investigated resting state activity by means of electroencephalography (EEG) among children with implanted cochlear implants showed considerable differences in effective connectivity during the resting state in children who developped good vs. poor language abilities after implantation (see Koirala et al.). Moreover, the study found good evidence that these differences can be detected and classified by support vector machine (SVM) algorithms which allowed the prediction of language and reading scores with high accuracy. Consequently, the two studies provide empirical evidence for the notion that human factors including interindividual differences in physiological states such as resting states provide a huge potential for BCI applications as predictors of BCI performance. Moreover, the results of the studies suggest that BCIs can be sensitive tools for the training of language competencies in patients with language or communicative impairments.

However, as outlined in the next section, there is still a research gap on linguistically and culturally sensitive non-ivasive BCI applications. This is despite increasing research in the field of neurocomputational language models (for an overview, see, for example, the joint Research Topic on *Neurocomputational Models of Language Processing*), and advances in the area of semantic reconstruction of continuous speech from non-invasive brain recordings (e.g., Tang et al., 2023).

### 4.3 Themes 4-5: language and culture sensitive BCI applications

Brain-computer interfaces are communication devices. As outlined in the perspective paper (Herbert) compiled in this section and published in the joint Research Topic on *Neurocomputational Models of Language Processing* (see Footnote 1), there are at least four levels at which language matters in a BCI system. First, at the level of the user, i.e., the user's language skills, competences and abilities. Second, at the signal input level, i.e., at the level of the paradigms used by the user for BCI communication. Third, at the interface design level, i.e., at the level of the spatial and temporal specifities of the language or symbols used for communication with the user including instructions and performance feedback. Fourth, at the signal preprocessing and decoding level, i.e., the stages of the BCI interface that are responsible for noise and artifact reduction, for extraction of the relevant information from the brain signal recordings and the classification of the relevant information from it for transfer and conversion of the signal into a reliable computer generated control signal. Systematic inter- and transdisciplinary research at all these four levels is required to design language sensitive BCI applications. Consequently, future BCI research should have a particular focus on human factors related to the language of the user to master the language diversity of BCI users worldwide. This would support the development of language sensitive applications for BCI-based language detection and training among vulnerable and healthy BCI users with different language competences and skills. Finally, given that language can vary across cultures, this means considering both linguistic and cultural differences in BCI use as important human factors for the development of BCI technology in future research (see Herbert for a detailed discussion). Moreover, as BCIs become more personalized, more privacy and ethical awareness will be required. As studies of speech BCI systems show, this also includes a detailed discussion of ethical issues in the use of artificial intelligence and user biases in the perception of computer-generated speech [for a discussion *The ethics of speech ownership in the context of neural control of augmented assistive communication* (see Footnote 2)].

The need to include language and cultural factors more systematically in BCI research is fostered by empirical evidence from neurolinguistics and cultural neuroscience suggesting linguistic and cultural variations in the perception, processing, and evaluation of stimuli. This is also demonstrated in another study published within this Research Topic. This study (Leshin et al.) dedicates its discussion to the interdependency of language, emotion and conceptual knowledge and provides empirical proof of this interdependency and its relationship with cultural differences. Using functional magnetic resonance imaging (fMRI) and an experiment during which participants with different cultural backgrounds were exposed to emotion category words before viewing faces expressing anger or disgust, this study could find differential functional connectivity between brain regions engaged in semantic retrieval, semantic processing, visual perception, and social cognition among participants with different cultural background. Consistent with previous research, these results show that even the presentation of simple words is sufficient to shape information processing and that this shaping is further modified by the participants' cultural differences.

In summary, the task of future research is to examine how these and other findings from affective and cognitive neurolinguistics and cultural neuroscience could be transferred to the field of BCI research in order to develop linguistically and culturally sensitive BCIs for the health promotion of healthy and disabled users (for a detailed discussion see Herbert). This also means discussing these topics in the context of future trends in the field of neurocomputational language models as well as in the context of privacy and ethics, which are currently the focus of additional joint Research Topics (see Footnotes 1, 2).

The present Research Topic *Analyzing and computing humans - the role of language, culture, brain and health* could represent a first step into this direction.

## Author contributions

CH: Conceptualization, Funding acquisition, Investigation, Methodology, Project administration, Supervision, Validation, Visualization, Writing – original draft, Writing – review & editing. GN: Writing – review & editing.

## Footnotes

^1^Research Topic: Neurocomputational Models of Language Processing. *https://www.frontiersin.org/research-topics/49147/neurocomputational-models-of-language-processing*.

^2^Research Topic: The ethics of speech ownership in the context of neural control of augmented assistive communication. *https://www.frontiersin.org/research-topics/50957/the-ethics-of-speech-ownership-in-the-context-of-neural-control-of-augmented-assistive-communication/authors*.
